# 

**Published:** 1884-09

**Authors:** 


### ORIGINAL COMMUNICATIONS:

Lecture on "Pericementitis" before the Dental Association of Northern New Jersey. Frank Abbott .... 475

The Possibilities of Hereditary Transmissions of Oral Lesions. W. C. Starbuck .... 483

Exposed Pulps. J. D. Patterson .... 487

Dental Education Once More. Faculty B. C. D. S. .... 491

### REPORTS OF SOCIETY MEETINGS:

Meeting of the Faculties of the Dental Colleges of the United States... 495

American Medical Association .... 507

Illinois State Dental Society .... 511

Chicago Dental Society .... 516

American Dental Association .... 520

Central Dental Association of Northern New Jersey .... 524

Southern Dental Association .... 528

### EDITORIAL:

The Educational Problem—Victory .... 531

Crowded Again .... 536

A Shocking Occurrence .... 536

Black's Micro-Organisms .... 537

Protective Association .... 538

Robinson's Fibrous Filling .... 538

An Appeal .... 539

Notice .... 539

Afflictive .... 539

Askings and Answers .... 540

